# A Potentially Useful Addition to Predict Spontaneous Resolution of Uterine Artery Pseudoaneurysm: Absence of Diastolic Flow

**DOI:** 10.1155/2018/2158248

**Published:** 2018-03-20

**Authors:** Yosuke Baba, Hironori Takahashi, Hiroyuki Morisawa, Daisuke Matsubara, Kohei Tamura, Rie Usui, Shigeki Matsubara

**Affiliations:** ^1^Department of Obstetrics and Gynecology, Jichi Medical University, Tochigi, Japan; ^2^Department of Pediatrics, Jichi Medical University, Tochigi, Japan

## Abstract

Pregnancy-associated uterine artery pseudoaneurysm (UAP) usually requires transarterial embolization (TAE) irrespective of the presence/absence of current bleeding. Some UAP cases spontaneously resolve without TAE; however, such UAP is not well characterized. Here, we suggest that Pulse Wave Doppler may become an addition to predict its spontaneous resolution. A woman underwent 1st-trimester vaginal termination. Vaginal bleeding repeated and, 36 days later, an intrauterine low-echoic mass (24 mm) with swirling blood flow and arterial waveforms (Pulse Wave Doppler) and an enhanced intrauterine sac-like structure without current extravasation were observed, leading to the diagnosis of UAP. Subsequently, the low-echoic mass mostly disappeared but the swirling flow was still observed, with Pulse Wave Doppler revealing arterial flow but the absence of diastolic flow. Finally, the flow disappeared and UAP resolved. This observation reconfirmed spontaneous UAP resolution. The “absent diastolic flow,” possibly indicative of decreased intrasac blood flow, may be a candidate for predicting UAP resolution.

## 1. Introduction

Delivery-/abortion-associated uterine artery pseudoaneurysm (UAP), when it ruptures, causes life-threatening hemorrhage. Transarterial embolization is usually performed, irrespective of whether UAP ruptures or not [1]. However, some UAP resolved [2, 3]. We also suggested that the absence of ultrasound-discernable low-echoic area (indicative of a UAP sac lumen) may predict the spontaneous resolution of UAP [4]. Rupture/nonrupture of UAP may depend on the balance between the intra-UAP blood flow/pressure and UAP wall strength, and thus, theoretically, blood flow of UAP may decrease before its resolution. Here, we propose that a unique feature of Pulse Wave Doppler, “absence of diastolic flow,” may predict spontaneous resolution of UAP.

## 2. Case Presentation

**Figure 1:**
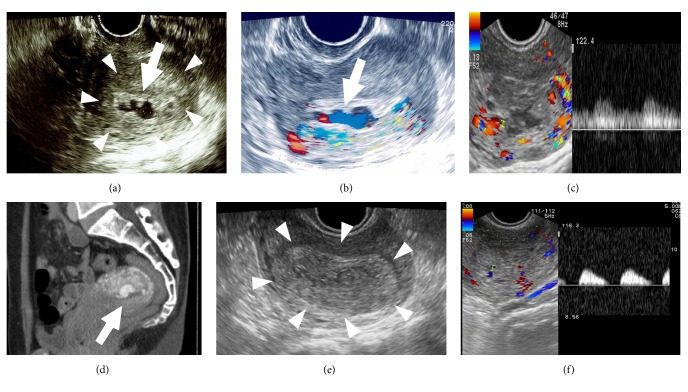
Findings of gray-scale ultrasound (a, e), Color Doppler (b), left panels of (c) and (f), Pulse Wave Doppler (right panels of (c) and (f)), and multiphase computed tomography (d). Images were taken on the first day of hospitalization (day 36 after vaginal termination) for (a)–(d) and day 55 for (e) and (f). (a) A low-echoic mass (arrow: size of 24 × 10 mm and indicative of an intrasac free lumen) is observed in the uterus, which is surrounded by a high-echoic mass (encircled by arrowheads: indicative of hematoma/thrombus). (b) Swirling blood flow (arrow) in the uterine cavity. (c) Swirling blood flow with arterial waveforms on Pulse Wave Doppler. At this stage, diastolic flow is evident. (d) An enhanced mass, measuring 24 mm in diameter, suggestive of a uterine artery pseudoaneurysm (arrow). (e) A high-echoic mass (arrowheads) is present but without an evident low-echoic mass. (f) Diastolic flow is absent on Pulse Wave Doppler, referred to as “absent diastolic flow” of the sac. This pattern was also confirmed by setting the “low-cut filter” lower. This “absent diastolic flow” was observed repeatedly and was also reproducible when the various scanning angles were changed.

A 43-year-old primigravida underwent pregnancy termination with gemeprost at the 13th gestational week. Thirty-six days later (day 36), she had vaginal bleeding. At her visit, she showed no active bleeding. Ultrasound revealed an intrauterine low-echoic mass/area (Figure 1(a)), in which Color Doppler revealed swirling blood flow (Figure 1(b)) and Pulse Wave Doppler revealed an arterial waveform (Figure 1(c)). Multiphase computed tomography confirmed an intrauterine sac-like structure with enhancement without extravasation from it (Figure 1(d)). We clinically diagnosed this condition as UAP. Small UAP, no current active bleeding, no current extravasation, and also her wish to do so helped us to adopt a “wait and see” strategy. On day 55, an intrauterine low-echoic area disappeared, replaced by the high-echoic mass/area (Figure 1(e)), but was still showing a Color Doppler flow. Pulse Wave Doppler revealed that systolic blood flow remained, whereas diastolic flow was absent (Figure 1(f)). On day 62, the swirling flow and arterial waveforms completely disappeared. On day 84, she vaginally expelled a blood clot, pathologically confirmed so. UAP did not recur. Patient anonymity was preserved and she consented to this publication.

## 3. Discussion

Some UAP cases resolve spontaneously; however, such UAP has not been well characterized. In this patient, during the course of UAP resolution, the grey-scale ultrasound-discernable low-echoic area became smaller but arterial blood flow remained: importantly, Pulse Wave Doppler revealed the “absence of diastolic flow” with systolic flow still remaining.

During the course of UAP resolution, an intra-UAP-sac thrombus may increasingly occupy the sac, making the free-blood-flow-containing space (low-echoic area) gradually smaller [2–5]. Systemic arterial flow is regulated by complex factors: cardiac output, preload blood flow, after-load including peripheral vessel resistance, arterial elasticity, and others. The pseudoaneurysmal-sac-flow regulation is much more complicated. Thus, although the mechanism of “absent diastolic flow” is unclear, the following may explain the phenomenon. While a thrombus occupies the sac, intrasac impedance increases, which decreases the total intrasac blood flow, with systolic flow remaining and diastolic flow being absent. Measuring actual blood flow is difficult but pattern recognition of “absent diastolic flow” is easy.

We previously reported three patients with spontaneously resolved UAP [3]: (1) all had a nontraumatic/slightly traumatic preceding procedure and (2) the UAP sac was small (10–15 mm in diameter). We also characterized UAP based on 50 consecutive angiographically confirmed cases [4]. The following three characterized current extravasation (-) (a low risk of rupture): (1) history of repeated bleeding, (2) no active current bleeding, and (3) indiscernible low-echoic intrauterine area. All these may indicate that hematoma/thrombus may gradually occupy the UAP sac and the rupture site is undergoing sealing. The present case fundamentally fulfilled all these five (2 + 3) characteristics.

The “absent diastolic flow,” possibly reflecting the decrease of intrasac blood flow, may predict spontaneous resolution of UAP. The term “absent end-diastolic velocity” of the fetal umbilical artery (a sign of fetal jeopardy) has gained popularity. Although the mechanism may differ between the two, this term may be easy to remember for obstetricians. Single case report cannot draw conclusion, and, thus, data accumulation is needed to confirm our present observation.
